# Carrier-Free Immobilization of Rutin Degrading Enzyme Extracted From *Fusarium* spp.

**DOI:** 10.3389/fbioe.2020.00470

**Published:** 2020-06-26

**Authors:** Yuan-Yuan Zang, Sha Yang, Yong-Qiang Xu, Zhi-Gang Chen, Tao Wu

**Affiliations:** ^1^Glycomics and Glycan Bioengineering Research Center, College of Food Science & Technology, Nanjing Agricultural University, Nanjing, China; ^2^College of Life Science and Engineering, Lanzhou University of Technology, Lanzhou, China; ^3^Department of Food Science, University of Tennessee, Knoxville, Knoxville, TN, United States

**Keywords:** *fusarium*, rutin degrading enzyme (RDE), cross-linked enzyme aggregate (CLEA), natural deep eutectic solvents (NADESs), enzymatic characteristics

## Abstract

In this study, a strain with rutin degrading enzyme (RDE) activity was screened from moldy tartary buckwheat and subsequently identified as *Fusarium* spp. The structure and enzyme characteristics of CLEA-RDE formed by immobilization via cross-linking were then investigated. Further, the optimal catalysis conditions of CLEA-RDE in natural deep eutectic solvents (NADESs) serving as hydrolysis solvents were also investigated. The results of SEM and spectrum indicated that CLEA-RDE became more stable than free-RDE due to the cross-linking. Interestingly, CLEA-RDE showed a wider range of pH adaptation and higher tolerance to low temperatures (20 – 30°C) and hydrophobic environments. The results of orthogonal experiments revealed that the optimal condition for rutin hydrolysis was under pH 5.0 and 40^o^C with the degradation rate of 10.65 mg min^−1^ L^−1^. The preparation of CLEA-RDE without a carrier-based immobilization method reduces the loss of enzyme activity, improves the stability of the enzyme and can be applied to the investigation of immobilization of various enzymes, thus providing a referred idea for the improvement of catalysts in industrial production.

## Introduction

Quercetin is a polyhydroxy flavonoid compound with a chemical name of 3, 3′, 4′, 5, 7-pentahydroxyflavone (Ulusoy and Sanlier, 2019) which is common in nature and is found in many foods such as onion, green tea and asparagus (Alinezhad et al., 2011; Nishimuro et al., 2015). It has various pharmacological effects, such as antioxidant effects (Simioni et al., 2018), preventing cardiovascular disease, and inhibiting the metabolism of cancer cells. In addition, quercetin is an efficacious substance in treating diabetes, neurological diseases, and obesity.

Rutin degrading enzyme (RDE), which can specifically convert rutin to quercetin, was first discovered by a Japanese scientist Yasuda Toshitaka from tartary buckwheat in 1993 (Yasuda and Nakagawa, 1994). Currently, chemical catalysis is normally used to obtain quercetin from rutin (Biesaga et al., 2007; Wach et al., 2007), which is not an environmentally benign process for such a low yield. In contrast, RDE catalysis is considered as a milder, greener and more efficient way to prepare quercetin from rutin. Tartary buckwheat (Yasuda and Nakagawa, 1994), *hypericum perforatum* (Biesaga et al., 2007), and onion (Turner et al., 2006) could be good sources of RDE.

Modern industry has been working to improve the stability of enzymes by immobilization (Liang et al., 2019). However, traditional immobilization methods are usually in need of an additional carrier which leads to a dilution or even loss of catalytic activity due to the complicated process (Bryjak and Kolarz, 1998; Tischer and Kasche, 1999). Therefore, carrier-free immobilization is beginning to get people's attention. Among them, CLEA has been proven to be a more effective method because it avoids the loss of origin activity, improves the catalytic efficiency (Cao et al., 2000) and stability to pH, temperature (Bian et al., 2019; Kulkarni et al., 2019; Talekar et al., 2020) and organic solvents (Cui et al., 2012, 2017; dong Cui et al., 2014; Cui and Jia, 2015; Bian et al., 2019; Razib et al., 2020). It is also easy to prepare and does not require high-purity enzymes, which means that different types of enzymes can be cross-linked at the same time (Mateo et al., 2004). There are two steps to prepare CLEA: Firstly, physical aggregates are obtained by changing the proximity between soluble enzyme molecules through changing the hydration state or altering the electrostatic constant of the solution through adding appropriate aggregation agents. Secondly, the cross-linking was performed using a bi-functional cross-linker to form CLEAs (Cao et al., 2003). So far, CLEA has been successfully used to immobilize many enzymes (Schoevaart et al., 2004; Talekar et al., 2012, 2014; Cui et al., 2016).

A previous study suggested that enzymes had good catalytic activity in non-aqueous or micro aqueous systems (Talekar et al., 2013). Recent studies found that the trace water preserved the active structure and improved the stability (Klibanov, 1986). Additionally, the substrate solubility was improved, and the side reactions were inhibited, which may have shifted the thermodynamic equilibrium to a desired direction (Zaks and Klibanov, 1984; Dordick, 1989). According to previous research (Zang et al., 2020), NADESs is a new kind of non-aqueous solvent, which inherits the excellent characteristics of ionic solvents and moreover process unique advantages of low toxicity, biodegradability, easy synthesis, and recycling.

In this study, strains containing RDE from moldy tartary buckwheat were screened. And we employed a novel, carrier-free immobilization method to form CLEA-RDE by altering the electrostatic constant of the solution and cross-linking through an addition of 25% glutaraldehyde solution. The activity and stability of this kind of enzyme were subsequently determined and compared with free-RDE. Finally, the effects of NADESs on promoting the catalytic activity of CLEA-RDE were also investigated.

## Materials and Methods

### Chemical and Biological Materials

The tartary buckwheat was purchased from a local market in Sichuan province, China. Rutin standard was obtained from Beijing Bailingwei Technology Co., Ltd. Bovine serum albumin, choline chloride, glycerol, ammonium sulfate, glutaraldehyde, tris, hydrochloric acid, dimethyl sulfoxide, methyl alcohol, ethyl alcohol, and trichloromethane were purchased from Aladdin Chemical Reagent Co., Ltd. (Shanghai, China). Ethidium bromide and DL2000 DNA Marker were purchased from Solarbio Life Science Co., Ltd. (Beijing, China). PCR amplification primers were synthesized by General Biosystems Co., Ltd. (Anhui, China). Agarose and gel cutting recovery kits were purchased from Sangon Biotech Co., Ltd. (Shanghai, China). DNA extraction kits were purchased from SparkJade Scientific Instruments Co., Ltd. (Shandong, China).

### Preparation of Major Reagents

#### Preparation of NADESs

NADESs that are suitable for RDE catalysis were selected from the literature (Zang et al., 2020). Choline chloride and glycerol were weighed at a molar ratio of 1:1 and then mixed at 200°C until a homogeneous liquid was formed. Then it was mixed with deionized water at a ratio of 80:20 (V/V) before use.

#### Preparation of Media

Acclimation medium: a liquid Czapek–Dox medium was modified by adding rutin (0, 2, 4, 6, 8, 10 g/50 mL) as a carbon source and correspondingly reducing the amount of sucrose accordingly (10, 8, 6, 4, 2, 0 g/50 mL).

Separation medium: solid Czapek–Dox medium.

Identification medium: Modified liquid Czapek–Dox medium by replacing sucrose with rutin, other ingredients remain unchanged.

### Source and Domestication of Strain

Organism suspension: weighed 10 g moldy buckwheat flour (mold at 25°C with a relative humidity of 50% for 3 days), dissolved in 200 mL of distilled water and then filtered for standby.

Inoculation: added 200 μL of suspension to sterilization medium.

Acclimation: Acclimate the strains in the medium with increasing rutin content. Five days is a period, and culture in a shaker at 28°C at a speed of 200 r/min. At the end of each cycle, 200 μL of the culture suspension was inoculated into the medium of next cycle, and the operation was repeated until the strain growth was stable.

### Isolation and Purification of Strains

Refer to GB 4789.16–2016 (GB 4789.16-2016, 2017), domesticated strains were isolated and purified by streak plate method, and then the plates were cultured at 28°C Repeated the above procedures until single strain was obtained and picked for liquid culture.

### Identification of Strain Activity

Two hundred Microliters of each organism solution was taken and inoculated in the identification medium, 28°C, 200 rpm for 3 d. Organism suspension was then centrifuged at 4000 r/min for 5 min, and the rutin amount in the supernatant was determined by HPLC as described in former study (Zang et al., 2020).

### Strain Preservation

The selected strain was inoculated and cultured at 30°C for 2–3 d, and then stored in a 4°C refrigerator.

### Strain Identification

#### Morphological Identification

Morphological observation: The isolated strains were inoculated into the Czapek–Dox medium with the spot planting method and cultured at 28°C for 5 days. During this period, the morphology of the colonies was observed and recorded.

Micro-structural observation: dropped a small drop of Lactobacillus carbonate cotton blue dye on the slide, took a small amount of mycelium and rinsed it in 50% ethanol solution, then washed the soaked mycelium with distilled water once, then immersed it in the dye solution, and covered the slide. They were observed under a 10, 20, 40, and 100 times optical microscope, and finally observed and recorded under 100 times oil microscope, for a preliminary identification of the organism, referring to Wei (1979).

#### Molecular biological identification

The purified strain was cultured on a flat plate with cellophane at 28°C for 3 d. The cultured fungal tissue was put into a mortar and ground into fine powder with liquid nitrogen. The DNA of the strain was extracted by SPARKeasy Fungus DNA kit.

PCR amplification of ITS rDNA target fragment was conducted as follows:

a) The primer sequence is:
Upstream primer ITS1: 5′-TCCGTAGGTGAACCTGCGG-3′;
Downstream primer ITS2: 5′-TCCTCCGCTTATTGATATGC-3′
b) PCR system
Ultra-pure water: 19 μL
2x Tap Master Mix: 25 μL
Primer 1: 2 μL
Primer 2: 2 μL
Substrate template: 2 μL DNA suspension
c) PCR reaction
  96°C pre-denaturation: 3 min
  $$\begin{array}{l} 96^{\circ }\text{C}\left(\text{denaturation}\right):45\text{ s}\\ 55^{\circ }\text{C}\left(\text{annealed}\right):45\text{ s}\\ 72^{\circ }\text{C}\left(\text{extension}\right):90\text{ s}\\ 72^{\circ }\text{C}\left(\text{extension}\right):8\;\min \end{array}\}35\text{ cycles}$$

Then, the PCR products were visualized on agarose gel (0.8% in TAE buffer). 0.24 g agarose powder was weighed and added to 30 mL 1 × TAE buffer, and the mixture was heated until boiling. After it had cooled, 1.5 μL ethidium bromide was added and mixed. The mixture was then poured into the template to form a glue. Then the glue was put into an electrophoresis tank. 1 × TAE buffer was added over the gel surface, the gel spotted in order, and the power turned on (130 V, 30 min). After running the gel, the ultraviolet imaging was observed in the gel imaging system.

The PCR products were then sent to General Biosystems Co., Ltd for strain identification. The ITS rDNA sequence of the isolated strain was compared with the ITS rDNA sequence recorded in NCBI by Blast, and strains with homology similarity above 95% were selected to construct a phylogenetic tree.

### Extraction and Purification of RDE From the Strain

The organism suspension above was centrifuged by refrigerated centrifuge at a speed of 8,000 × g for 45 min, the supernatant was stored at 4°C as crude enzyme.

The crude enzyme solution was precipitated by ammonium sulfate, then ammonium sulfate was slowly added to 60% saturation, stirring until completely dissolved and resting overnight. After the refrigerated centrifugation at a speed of 8000 × g for 45 min, the precipitate was discarded and the supernatant was then slowly added with ammonium sulfate to 90% saturation, stirred until dissolved and rested overnight. The above refrigerated centrifugation (8,000 × g, 45 min) was repeated, the supernatant discarded, and the precipitate added to 60 mL acetic acid buffer solution (0.02 M, pH=5) to make a suspension. Finally, the suspension was transferred to a dialysis bag (shanghai yuanye Bio-Technology Co., Ltd. interception molecular weight of 4,000) and dialyzed against 4°C distilled water overnight.

### Enzyme Activity Determination

#### Enzyme Activity Determination of Free-RDE

The crude enzyme was taken as free-RDE, 55 μL of which was added to 1 mL of 80% ChGly-water containing rutin (1 mg mL^−1^) and adjusted the pH to 7. The mixture was incubated at 37°C for 15 min. The reaction was stopped by adding 1 mL of methanol. The amount of degraded rutin was determined by HPLC as described in a former study (Zang et al., 2020).

One unit of RDE activity was defined as the amount of enzyme required to catalyze 1 μg rutin per min under the conditions above.

#### Protein Content Determination of Free-RDE

The protein content of free-RDE was determined using the Bradford method, BSA was used to prepare standard solution (0.005–0.025 mg mL^−1^), its UV absorbance at 595 nm was measured to establish a standard curve.

#### Enzyme Activity Determination of CLEA-RDE

The enzyme activity of CLEA-RDE was determined by the above method. The enzyme activity unit of CLEA-RDE was also defined as above.

Recovery rate of CLEA-RDE activity (%) = CLEA-RDE activity / total activity of free-RDE added × 100%.

### Preparation and Optimization of CLEA-RDE

#### Preparation of CLEA-RDE

0.1 μg bovine serum albumin (BSA) was slowly added in 4.9 mL of dialyzed enzyme solution diluted 20-fold with acetate buffer (0.02 mol L^−1^, pH=5) for precipitation, and rested at room temperature for 30 min. Twenty-five percentage glutaraldehyde solution was then slowly added to a concentration of 0.5% (v/v). Cross-linking was applied at 20°C for 3 h with continuous gentle stirring, and after being centrifuged (4° C, 8,000 × g, 45 min) the precipitates were washed by acetate buffer (0.02 mol L^−1^, pH=5) 3 times and were collected and then suspended with a 4 ml Tris-HCl buffer.

#### Optimization of Immobilization Conditions of CLEA-RDE

Six factors were selected, namely: cross-linking agent concentration (0.5%, 1%, 1.5%, 2%, v/v), pH (4, 5, 6, 7, 8, 9), time (1, 2, 3, 4 h), BSA amount (0, 0.5, 1, 5, 10 μg), dilution factor of enzyme solution (0, 20, 50, 100, 200), and temperature (5, 20, 30, 40, 50°C). These were tested one factor at a time, and the rutin degraded amount was determined using the method above.

Based on the results of the single factor test (Figure S1), an orthogonal L9 (3)^3^ test was designed (Table S1) to optimize the immobilization conditions of CLEA-RDE. Factors were dilution factor of the enzyme solution, temperature, pH and cross-linking agent concentration. The immobilized enzyme activity was the dependent variable.

### Structure Characterization of CLEA-RDE

The surface structure of the freeze-dried CLEA-RDE and free-RDE was observed under a vacuum with a S-4800 field emission scanning electron microscope (Hitachi, Ltd.). The infrared spectrum changes of RDE before and after cross-linking were analyzed in the range of wavelength 4,000–400 cm^−1^ with a resolution of 4, scanning time of 32 by IR200 Fourier transform infrared spectrometer (FTIR) (Nicolet Co., Ltd.). A L20A UV spectrophotometer (SHIMADZU Co., Ltd.) was used to scan the RDE at full wavelength to analyze the change of the maximum absorption wavelength.

### Optimization of Hydrolysis Conditions of CLEA-RDE

As a protein, the catalytic activity of CLEA-RDE is often affected by the temperature, time and pH of the reaction system. At the same time, the ratio of enzyme and reaction substrate in the system also affects the yield of the hydrolysis reaction. After immobilization, the structural and physicochemical properties of the enzyme protein were changed compared to the free enzyme, and the optimal reaction conditions need to be redesigned to explore the experiment.

CLEAs amount (55, 100, 150, 200 μL), pH (4, 5, 6, 7, 8, 9), time (5, 15, 30, 45, 60 min), and temperature (20, 40, 60, 80°C) were selected to establish single factor experiments. One factor at a time was tested, and the rutin degraded amount was determined using the method above.

According to the results of the single factor tests (Figure S2), 3 factors (time, pH, temperature) were selected for further optimization. The immobilized enzyme activity was the dependent variable. The design of the orthogonal experiment is shown in Table S2.

### Study on Enzymatic Properties of CLEA-RDE

#### Effect of Temperature on the Catalytic Activity of CLEA-RDE

Determination of optimum reaction temperature: the activity determination was performed at 20, 30, 40, 50, 60, and 70°C.

Determination of the thermal stability of enzymes: the enzyme was incubated at 20, 30, 40, 50, 60, and 70°C for 2 h before activity assay.

#### Effect of pH on the Catalytic Activity of CLEA-RDE

Determination of optimum reaction pH: the enzyme activity was determined in the system with pH 4–8.

Determination of the pH stability of enzymes: the enzyme was incubated in the system of pH 4–8 for 2 h before activity assay.

#### Effect of Organic Solvents on the Catalytic Activity of CLEA-RDE

Eighty percentage dimethyl sulfoxide, methanol, ethanol and chloroform were used to determine the activity of RDE before and after cross-linking, and the enzyme activity recovery rate was calculated.

#### Determination of Enzymatic Kinetic Parameters

Catalytic rate of free-RDE and CLEA-RDE was determined with 0.1, 0.2, 0.3, 0.4, 0.5, and 0.6% substrate solutions at the time of 1.5 min by the way above. 1/[s] and 1/[r] were taken to establish a coordinate system, draw a Lineweaver–Burk curve, and calculate *K*_m_ and V_max_ from the slope and intercept.

#### Reuse and Storage Stability of CLEA-RDE and Free-RDE

The stability of CLEA-RDE during batch reactions was investigated. After each batch reaction (reaction conditions: 55 μL CLEA-RDE; 1 mL 80% ChGly-water containing 1 mg mL^−1^ rutin; pH=7; 37°C; 15 min), the catalyst was recovered by filtration and used again in a fresh reaction mixture to determine the conversion. The residual activity of each batch was calculated by setting the enzyme activity of the first batch as 100%.

The storage stability of CLEA-RDE and free-RDE were determined at 25°C and 4°C. The initial activity of CLEA-RDE determined just after preparation was set as 100%. The activity was measured every month.

## Result and Discussion

### Strain Screening Results

#### Identification of Strain Activity

A strain of fungus (F1) and a strain of bacteria (B1) were domesticated with the conversion of 53.9 and 2.4%, respectively. Thus, strain F1 with high catalytic activity was further identified and used as the subject of subsequent experimental research.

#### Morphological and Molecular Biology Identification

The colony identification was obtained from Figure S1A. The strain F1 was round on the culture medium, with flat colonies and cotton-fiber-shaped white hyphae. Microscopic observation was displayed in Figure S1B. The mycelium had a septum and branches, producing long tube-shaped arthrospores. According to GB 4789.16-2016 (GB 4789.16-2016, 2017), the strain was initially identified as *Fusarium* spp, and the aerial hyphae could develop on potato-glucose agar or Czapek–Dox medium, with various forms of megaconidia such as sickle, linear, and spindle.

The PCR product of strain F1 was verified using 0.8% agarose gel electrophoresis. Figure S2 showed that the amplified product had a single band and a fragment size of about 600 bp. The band was clear and suitable for sequencing experiment.

According to the sequencing results (Zang et al., 2020) (Figure S3), F1 was identified as *Fusarium* spp.

### Orthogonal Experiments to Optimize Immobilization Conditions

CLEA-RDE with an enzyme activity of 3.84 mg min^−1^ L^−1^ was obtained through immobilization, and its activity recovery rate was 20.36%. To optimize the immobilization conditions, single factor test was set up. Orthogonal experiments were designed according to the single factor test (Figure S4). Glutaraldehyde was the most commonly used cross-linking agent in the preparation of CLEAs which greatly affected the immobilization rate (Razib et al., 2020). The cross-linking of glutaraldehyde to the enzyme was mainly through the covalent bonding between the aldehyde groups at both ends and the α-lysine on the surface of the enzyme molecule to obtain insoluble cross-linked enzyme aggregates. When the concentration of the cross-linking agent was too low, cross-linking failed to occur; when the concentration was too high, cross-linking between enzyme molecules and intra-molecules closely interconnected, and glutaraldehyde might even bind to the enzyme active center, resulting in a decrease in enzyme activity. The effect of cross-linker concentration on immobilization was presented in Figure S4A. It could be seen that the immobilization efficiency was the highest when the glutaraldehyde concentration is 0.5%.

As shown in Figure S4B, the immobilization efficiency was the highest at pH 7, because the spatial structure, active site and binding site dissociation state of the enzyme varied greatly under different pH environments. At pH 7, it was the most suitable environment for RDE to form cross-linking with glutaraldehyde.

The effect of different cross-linking time on the immobilization efficiency was displayed in Figure S4C. The immobilization efficiency reached the maximum at 3 h, then excessive cross-linking occurred and the enzyme activity decreased.

The effect of the BSA addition on the immobilization efficiency was depicted in Figure S4D. The immobilization efficiency was the highest when the concentration of BSA was 1 mg mL^−1^, and then decreased with the increment of BSA concentration. It has been proved that the addition of BSA can provide the amino acid groups required for complete cross-linking, thus improving the activity, stability (Cui et al., 2012) and separation of CLEA (Razib et al., 2020), But notably, excess BSA would compete with RDE and inhibit the cross-linking. Results in this study were also consistent with this conclusion.

Figure S4E showed that the amount of enzyme added also had an effect on the enzyme activity. The activity of cross-linking enzyme increased first with the increasing amount of enzyme, and then decreased after reaching the maximum value. This was because the number of cross-linking groups of glutaraldehyde was limited, after the saturation value was exceeded, the amount of enzyme continued to increase, resulting in a decrease in immobilization stability. At the same time, an increase in the concentration of the enzyme also caused an increase in the volume of the aggregate to cause diffusion restrictions, leading to the difficult combination between the substrate and enzyme active center and therefore reducing the enzyme activity. Here the best immobilization efficiency was achieved when the dilution ratio of the enzyme solution is 50 times.

Figure S4F demonstrated that the immobilization efficiency was the highest at 20°C, and the temperature affected the molecular structure of the protein and the cross-linking of the enzyme.

According to the results above, dilution factor of the enzyme solution, temperature, pH and cross-linking agent concentration were further optimized (Table S1). The results were shown in Table 1. As presented in Table 1, the factors affecting the immobilization of RDE were as follows, with a decreasing order: cross-linking agent concentration, dilution factor of the enzyme solution, pH and temperature. Combined with the results of the single factor test, the optimal immobilization conditions were: the crude enzyme solution was diluted for 20 times, 0.1 μg BSA added, stood at room temperature for 30 min, and then 25% glutaraldehyde solution slowly added to a final concentration of 0.25%. The pH of the action system was altered to 6, and cross-linked at 4°C for 3 h with continuous gentle stirring. After the centrifugation, the precipitates were washed and dissolved to obtain CLEA-RDE. Three parallel experiments were performed with a degradation rate of 8.16 mg min^−1^ L^−1^.

**Table 1:** Design and results of the L9 (3^4^) orthogonal test^a^.

	**Dilution factor of the enzyme solution**	**Temperature (°C)**	**pH**	**Cross-linking agent concentration**	**Degradation rate** **(mg min^**−1**^ L^**−1**^)**
1	20	4	6	0.25%	8.01
2	20	20	7	0.5%	5.00
3	20	30	8	0.75%	4.59
4	50	4	7	0.75%	4.70
5	50	20	8	0.25%	6.49
6	50	30	6	0.5%	5.37
7	100	4	8	0.5%	3.91
8	100	20	6	0.75%	4.43
9	100	30	7	0.25%	6.12
K_1_	5.87	5.54	5.94	6.87	–
K_2_	5.52	5.31	5.27	4.76	–
K_3_	4.82	5.36	5.00	4.58	–
R_j_	1.05	0.23	0.94	2.30	–

a *Standard degradation conditions: 55 μL CLEA-RDE, 1 mL 80% ChGly with 1 mg mL^−1^ rutin content, 37°C, 15 min*.

### Structural Characterization of CLEA-RDE

**Figure 1:**
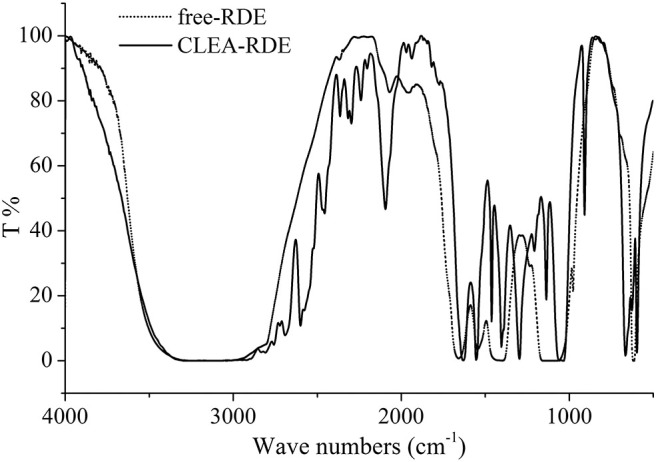
FTIR results of free-RDE and CLEA-RDE.

According to the FTIR results from Figure 1, there was a strong peak at 1500 cm^−1^ of CLEA-RDE compared to free-RDE, which indicated that there were more amide bonds formed by the reaction of amino groups and aldehyde groups in the aggregate. It verified that CLEA-RDE formed a tightly structured cross-linked enzyme aggregate through the cross-linking of glutaraldehyde.

**Figure 2:**
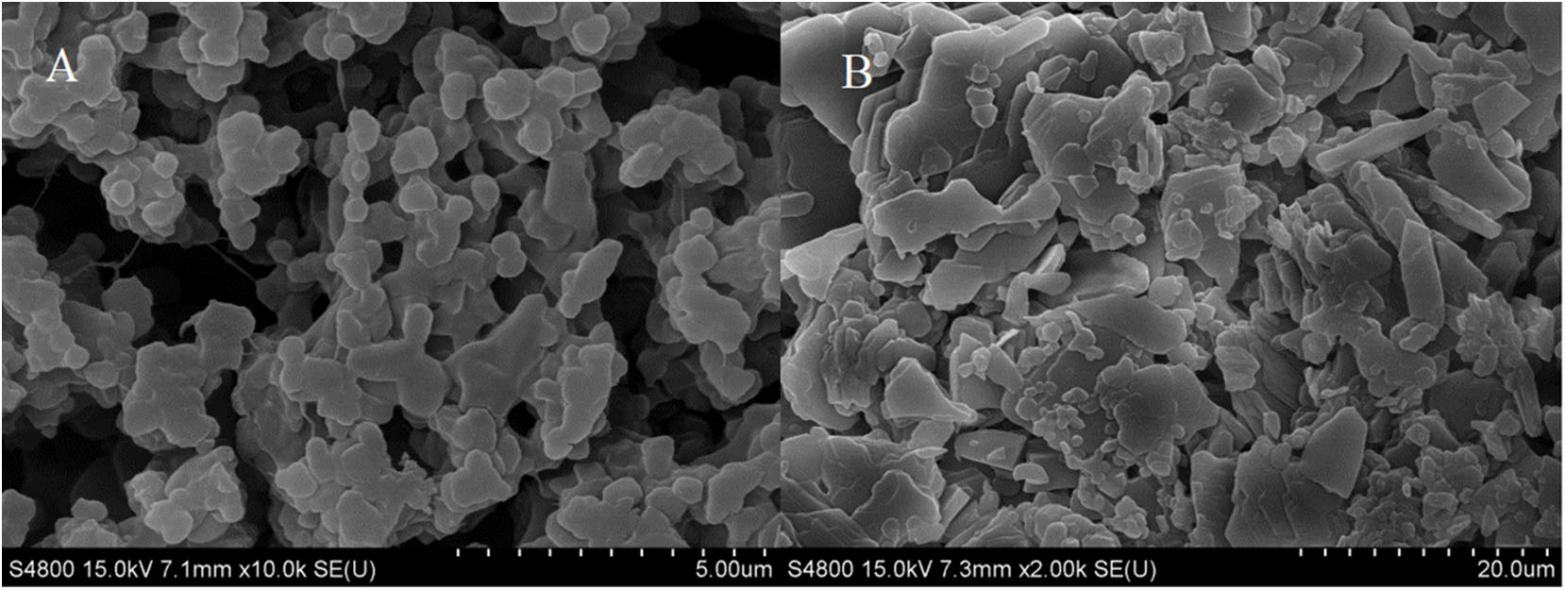
SEM of RDE before and after aggregation [**(A)** RDE before cross-linking; **(B)** CLEA-RDE].

Schoevaart et al. (2004) conducted an SEM of the structure of 12 kinds of CLEAs and found that the structure of different enzyme aggregates could be divided into two types: I highly hydrophobic with no glycosylation on the surface, II highly hydrophilic and glycosylated on the surface. From Figure 2, we could figure out that the RDE had significant structural differences before and after cross-linking: the RDE had a large regular spherical structure before cross-linking; after cross-linking, it formed a structure between I and II that was neither a ball nor belonging to a random cross-linked structure. However, the addition of the cross-linking agent caused covalent bonding in the aggregates, and the stability and mechanical strength of the RDE were improved.

**Figure 3:**
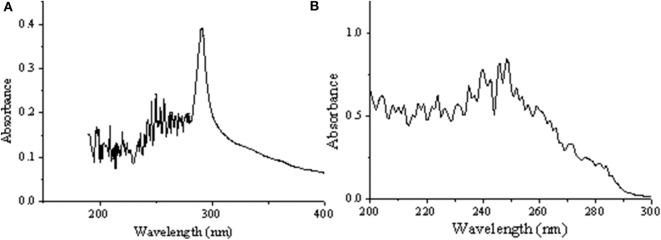
UV full wavelength scanning of free-RDE and CLEA-RDE [**(A)** free-RDE; **(B)** CLEA-RDE].

Figure 3 displayed that the maximum absorption wavelength of RDE changed significantly before and after cross-linking. The maximum absorption wavelength of free-RDE (protein content 21.93 μg mL^−1^) was 290 nm, a characteristic absorption peak of protein, while the maximum absorption wavelength of CLEA-RDE moved toward the short-wave direction. It was speculated that this blue shift might be caused by the introduction of a conjugated system into the amide bond.

### Orthogonal Experiments to Optimize Hydrolysis Conditions

According to Figure S5A, when the amount of CLEA-RDE was in the range of 55 – 200 μL, the amount of rutin degradation decreased with the increasing amount of enzyme.

The effect of pH value in the reaction system on the amount of rutin degradation could be seen in Figure S5B. In the range of pH 4 – 9, the amount of rutin degradation increased first and then decreased as the pH was increasing. The maximum value appeared at pH 6, which meant that under the reaction system, the RDE had the highest enzyme activity at pH 6.

The effect of reaction time on the amount of rutin degradation was described in Figure S5C. With the increase of the reaction time, the amount of degradation increased, and the maximum value remained stable at about 30 min, that is, the reaction was completed in this reaction system.

Temperature is one of the important factors affecting the activity of enzyme protein. As shown in Figure S5D, in the range of 40 – 80°C, the maximum amount of rutin degradation appeared at about 60°C as the temperature increased, and then the enzyme activity began to decrease. That meant in this study, 60°C was the optimal temperature for CLEA-RDE to exert enzyme activity in the experimental system described herein.

Orthogonal experiments were designed according to the single factor experiment (Table S2). The reaction time, pH, and temperature were further optimized, and the degradation amount of rutin were used as the comprehensive inspection index. The optimization results were shown in Table 2. From the R_j_ value in Table 2, the influence of various factors on the experimental results could be obtained. The pH of the reaction system had the largest effect on the degradation of rutin, followed by temperature, and the reaction time had the least effect on the catalytic yield. According to the K value, the optimal level combination under each factor was selected as the optimal reaction conditions: the pH of the reaction system was 5, the reaction time was 15 min, and the reaction temperature was 40°C. Under the optimal conditions, three verification tests were performed to obtain a degradation rate of 10.65 mg min^−1^ L^−1^.

**Table 2:** Design and results of the L9 (3^3^) orthogonal test^a^.

	**Time (min)**	**pH**	**Temperature**	**Degradation rate**
			**(^**°**^C)**	**(mg min^**−1**^ L^**−1**^)**
1	15	5	40	8.58
2	15	6	50	4.39
3	15	7	60	1.11
4	30	5	50	4.81
5	30	6	60	0.30
6	30	7	40	2.53
7	45	5	40	3.56
8	45	6	60	1.49
9	45	7	50	1.17
K̄_1_	4.69	5.65	4.89	–
K̄_2_	2.55	2.06	3.46	–
K̄_3_	2.07	1.60	0.97	–
R_j_	2.62	4.05	3.92	–

a *Standard degradation conditions: 55 μL CLEA-RDE, 1 mL 80% ChGly with 1 mg mL^−1^ rutin content, 37°C, 15 min*.

### Study on Enzymatic Properties of CLEA-RDE

#### Thermal Properties of CLEA-RDE

**Figure 4:**
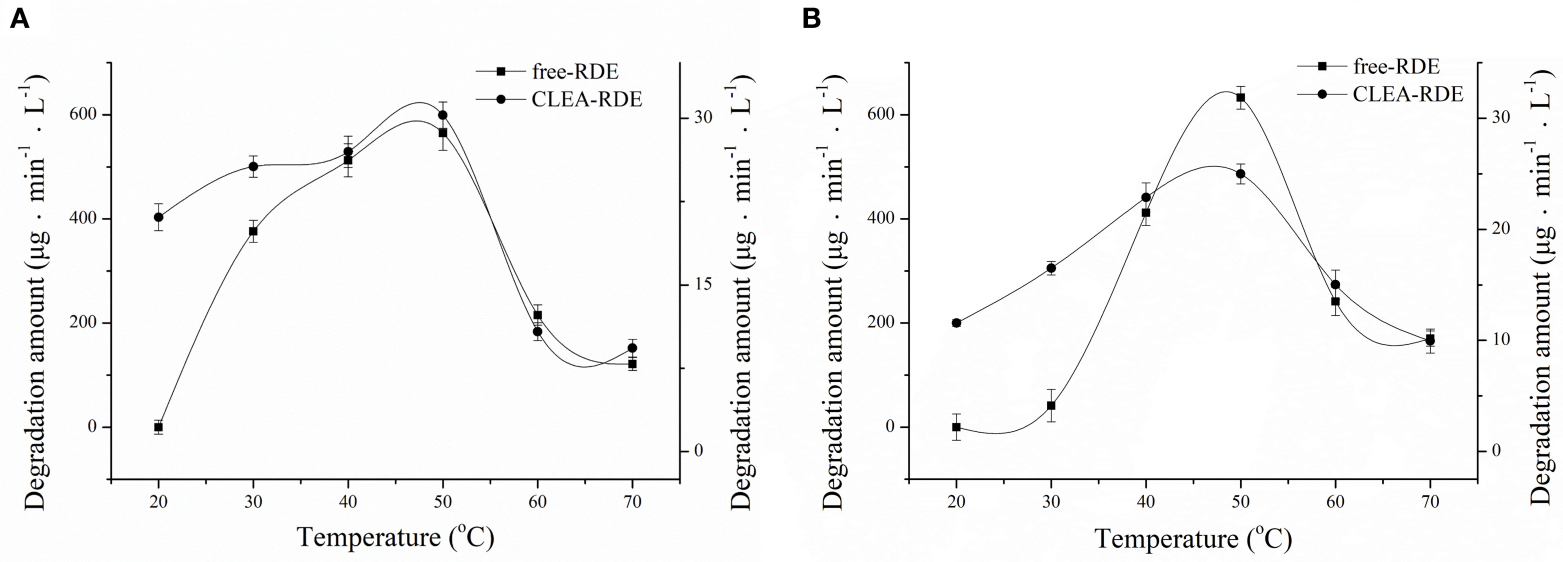
Effect of temperature on the stability **(A)** and activity **(B)** of free-RDE and CLEA-RDE. Standard degradation conditions: 55 μL CLEA-RDE or free-RDE solution, 1 mL 80% ChGly with 1 mg mL^−1^ rutin content, 37°C, 15 min.

The comparison of the thermal stability of CLEA-RDE and free-RDE was described in Figure 4A. CLEA-RDE remained stable in the temperature range of 20 to 50°C, and notably, the stability was improved at low temperature (20 – 30°C) compared with the free-RDE. And from Figure 4B, the optimal temperature of CLEA-RDE and free-RDE were not much different, both about 50°C. These results demonstrated that the thermal properties of CLEA-RDE were improved compared to free-RDE, which was also confirmed in the cross-linked enzyme aggregates of penicillin acylase (Pchelintsev et al., 2009).

#### pH Properties of CLEA-RDE

**Figure 5:**
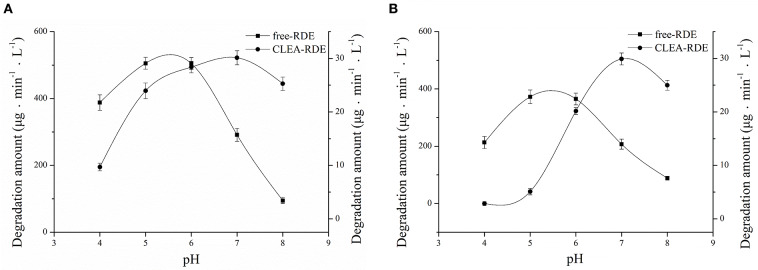
Effect of pH on the stability **(A)** and activity **(B)** of free-RDE and CLEA-RDE. Standard degradation conditions: 55 μL CLEA-RDE or free-RDE solution, 1 mL 80% ChGly with 1 mg mL^−1^ rutin content, 37°C, 15 min.

As Figure 5A showed, RDE could maintain stability in a wide range of pH and its resilience to alkaline environments was greatly improved. According to Figure 5B, the most favorable pH of the CLEA-RDE was 7, and it moved to the direction of alkali compared with the free-RDE (pH=5), which was due to the decrease in the conformational flexibility of enzyme explained in former study (Talekar et al., 2013).

#### Stability of CLEA-RDE to Organic Reagents

From Table 3, it could be obtained that as the LogP value of the organic reagent increasing, the remaining enzyme activity of CLEA-RDE and free-RDE both increased. But the CLEA-RDE was higher, especially in hydrophobic solvents, and the residual activity of CLEA-RDE (121.7%) was almost twice that of free-RDE (66.3%) in ethyl alcohol. These results indicated that the RDE immobilized by cross-linking method had a higher stability in a hydrophobic environment, which was consistent with the cross-linked enzyme aggregates of penicillin acylase (Pchelintsev et al., 2009) and phenylalanine ammonia lyase (Cui et al., 2012).

**Table 3:** Stability of CLEA-RDE and free-RDE in different organic reagents^a^.

**Organic solvents**	**LogP**	**Residual enzyme activity**	**Residual enzyme activity**
		**of CLEA-RDE (%)**	**of free-RDE (%)**
Dimethyl sulfoxide	−1.3	6.26	2.68
Methyl alcohol	−0.76	9.46	7.96
Ethyl alcohol	−0.24	121.68	66.32
Trichloromethane	2.0	180.01	102.43

a *Standard degradation conditions: 55 μL CLEA-RDE or free-RDE solution, 1 mL 80% ChGly with 1 mg mL^−1^ rutin content, 37°C, 15 min*.

#### Determination of Enzymatic Kinetic Parameters

Enzymatic kinetic parameters were critical characteristic data in enzymatic and metabolic studies. The *K*_m_ value was constant for a particular enzyme. The larger the *K*_m_ value, the smaller the affinity of the enzyme and the substrate; otherwise, the greater the affinity.

**Figure 6:**
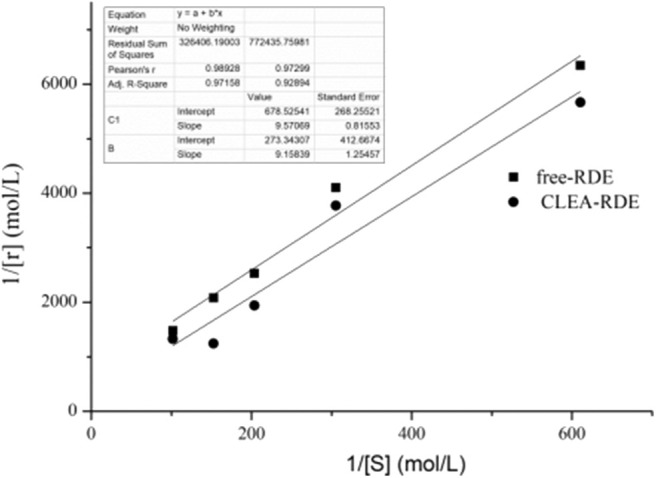
Lineweaver-Burk plot of free-RDE and CLEA-RDE. Standard degradation conditions: 55 μL CLEA-RDE or free-RDE solution, 1 mL rutin-80% ChGly of different rutin concentration. 37°C, 1.5 min.

As shown in Figure 6, compared with free-RDE (*K*_m_ = 1.6 mM, V_max_= 15 mM), the *K*_m_ value of CLEA-RDE (*K*_m_= 34 mM, V_max_= 3.7 mM) increased, the V_max_ value decreased, and the decrease in affinity with the substrate might be due to the fact that the active center of the CLEA-RDE was covered and the binding to the substrate was hindered.

#### Reuse and Storage Stability of CLEA-RDE and Free-RDE

In this study, CLEA-RDE could be used at least 8 times without significant loss of activity (96.2%), which indicated its strong operational stability.

The storage stability of CLEA-RDE and free-RDE were determined and 3 months later, both free-RDE (94.3%) and CLEA-RDE (95.7%) maintained their original activities. The results showed that CLEA-RDE could maintain its high catalytic activity during storage.

## Conclusions

In this experiment, a new source of RDE was explored by microbial utilization of tartary buckwheat and CLEA-RDE was successfully prepared based on a carrier-free immobilization method. NADESs was proved to be useful for maintaining enzyme activity and stability. Under the optimal reaction conditions (in 1 mL of rutin concentration of 1 mg mL^−1^ 80% ChGly-water co-solvent, 55 μL of CLEA-RDE were added, and the reaction was performed at 40°C for 15 min), the rate of rutin degradation reached 10.65 mg min^−1^ L^−1^. In addition, this carrier-free immobilization way for the preparation of CLEA-RDE was critical to prevent the enzyme activity from loss. Hence these results would have great value for the development of the catalyst manufacturing industry.

## Data Availability Statement

The datasets generated for this study can be found in the NCBI SUB7018537 F1 MT102379.

## Author Contributions

Y-YZ and Z-GC contributed conception and design of the study. Y-YZ and SY organized the database and performed the statistical analysis. Y-YZ wrote the first draft of the manuscript. TW and Y-QX contributed to manuscript revision, read, and approved the submitted version.

## Conflict of Interest

The authors declare that the research was conducted in the absence of any commercial or financial relationships that could be construed as a potential conflict of interest.
