# Supplementary material for: Carrier-Free Immobilization of Rutin Degrading Enzyme Extracted From Fusarium spp
Source: Front Bioeng Biotechnol. 2020 Jun 26;8:470. doi: 10.3389/fbioe.2020.00470 (PMC7332768; doi:10.3389/fbioe.2020.00470)
Supplement: Supplementary file 1 [file Data_Sheet_1.docx]

***Supplementary Material***

## Morphological observation of strain


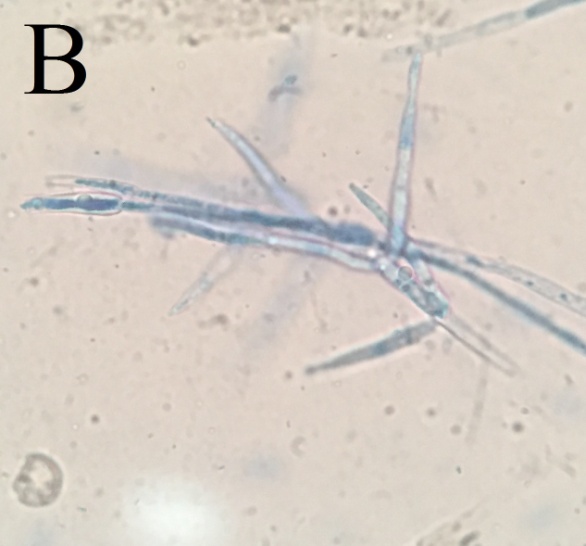

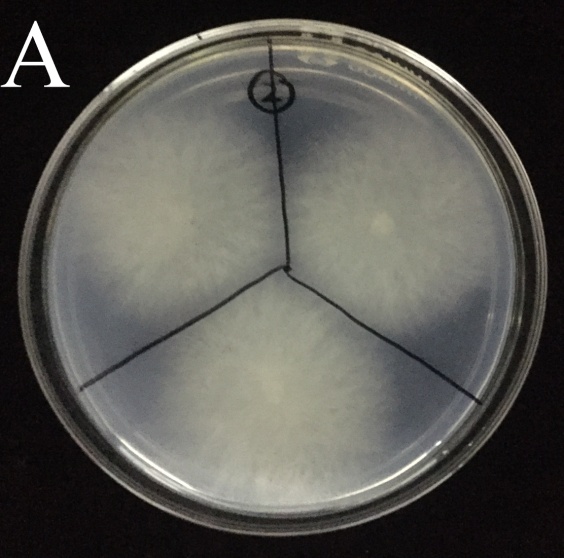


**Figure S1** Morphological identification of strains F1 (**Figure S1A** Observation of colony morphology; **Figure S1B** Microscopic observation)

## 0.8% agarose gel electrophoresis of F1 PCR products


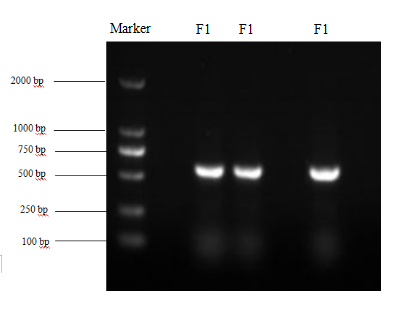


**Figure S2** Electrophoresis verification of strains F1

## Phylogenetic tree construction of F1


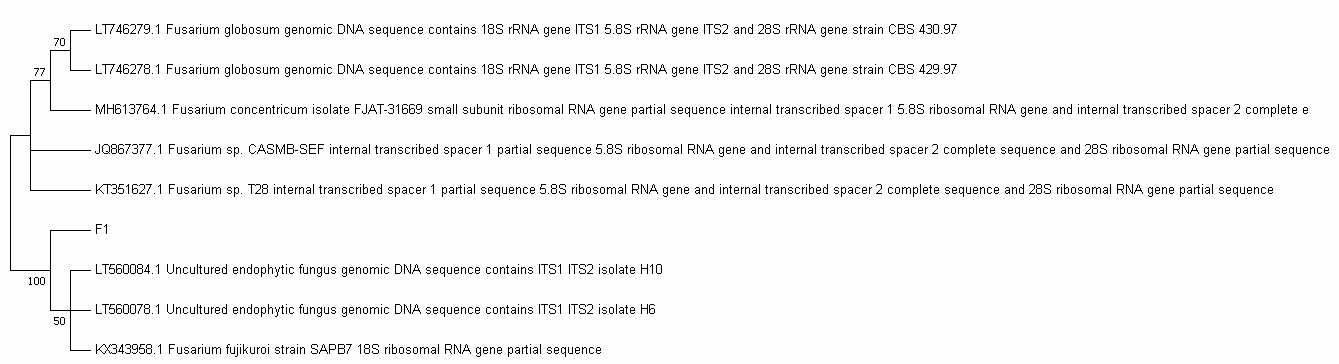


**Figure S3** Phylogenetic tree of F1 strain constructed based on 16s rDNA gene sequence

## Single factor experiment to optimize immobilization conditions

















**Figure S4** Single factor experiment on the eﬀect of cross-linking agent concentration (**Figure S4 A**), pH (**Figure S4 B**), time (**Figure S4 C**), BSA amount (**Figure S4 D**), dilution factor of enzyme solution (**Figure S4 E**) and temperature (**Figure S4 F**) on the immobilization eﬃciency of RDE. Standard degradation conditions: 55 *μ*L rutin degrading enzyme aggregate, 1 mL 1 mg / mL rutin-80% ChGly, 37 ^o^C , 15 min.

## Orthogonal experiments to optimize immobilization conditions

**Table S1**. Design of L9 (3^3^) orthogonal test.

|  | Dilution factor of the enzyme solution | Temperature  (^o^C) | pH | Cross-linking agent concentration |  |
| --- | --- | --- | --- | --- | --- |
| 1 | 20 | 4 | 6 | 0.25% | |
| 2 | 50 | 20 | 7 | 0.5% | |
| 3 | 100 | 30 | 8 | 0.75% | |

## Single factor test to optimize hydrolysis conditions












**Figure S5** Single factor experiment on the eﬀect of CLEAs amount (**Figure S5 A**), pH (**Figure S5 B**), time (**Figure S5 C**), and temperature (**Figure S5 D**) on the degradation eﬃciency of RDE. Standard degradation conditions: 55 *μ*L rutin degrading enzyme aggregate, 1 mL 1 mg / mL rutin-80% ChGly, 37 ^o^C , 15 min.

## 6. Orthogonal experiments to optimize hydrolysis conditions

**Table S2**. Design of L9 (3^4^) orthogonal test^a^.

|  | Time (min) | pH | Temperature (^o^C) |
| --- | --- | --- | --- |
| 1 | 15 | 5 | 40 |
| 2 | 30 | 6 | 50 |
| 3 | 45 | 7 | 60 |
